# Robotic single-site excision of ovarian endometrioma

**DOI:** 10.1186/s40738-015-0011-4

**Published:** 2015-12-21

**Authors:** Antonio R. Gargiulo, Colleen Feltmate, Serene S. Srouji

**Affiliations:** 1 Division of Reproductive Endocrinology and Infertility, Brigham and Women’s Hospital, Harvard Medical School, 75 Francis St., Boston, MA USA; 2 Division of Gynecologic Oncology, Brigham and Women’s Hospital, Harvard Medical School, 75 Francis St., Boston, MA USA; 3 Center for Robotic Surgery, Brigham and Women’s Hospital, Harvard Medical School, 75 Francis St., Boston, MA USA

**Keywords:** Endometrioma, Endometriosis, Robotic surgery, Single-incision laparoscopic surgery

## Abstract

**Background:**

Conventional single-incision laparoscopic surgery has been successfully employed for treatment of ovarian endometriomas. However, this technique presents surgeons with formidable ergonomic challenges, that make its widespread adoption unlikely. Robotic assistance in single-incision laparoscopic surgery provides adequate instrument triangulation through a single fulcrum, while eliminating ergonomic challenges to the surgeon. The objective of this video is to describe a novel technique of laparoscopic excision and ablation of ovarian endometriomas with single-site robotic assistance. Footage from a single surgical case is shown in our video. The da Vinci Si surgical system with da Vinci Single-Site platform was utilized. A flexible CO2 laser fiber was employed as the main energy tool.

To describe a technique of single-incision laparoscopic excision and ablation of endometriomas with robotic assistance.

Footage from a single surgical case is shown in this video. The da Vinci Si surgical system with da Vinci Single-Site platform was utilized. A flexible CO2 laser fiber was employed as the main energy tool.

**Results:**

Our technique achieved excellent surgical, clinical and cosmetic results, with complete excision and ablation of the endometriomas and no complications. The procedure was completed in day-surgery setting.

**Conclusion:**

Our step-by-step video tutorial shows how the dedicated single incision laparoscopy technology for the da Vinci Si surgical system can be safely and effectively applied to the excision and ablation of ovarian endometriomas.

**Electronic supplementary material:**

The online version of this article (doi:10.1186/s40738-015-0011-4) contains supplementary material, which is available to authorized users.

## Background

Endometriomas are peculiar ovarian pseudocysts that lack a true capsule and originate from the invagination and distension of an area of the ovarian cortex. Endometriomas can negatively impact ovarian reserve, cause pain, and can also raise the suspicion of ovarian malignancy. However conservative surgical intervention may be indicated in women of reproductive age. Surgical removal of endometriomas has been associated with significant loss of ovarian follicular reserve and even premature ovarian failure [1]. Therefore, safe and reproducible techniques of endometrioma excision are of paramount clinical importance [2]. Conventional single-incision laparoscopic surgery has been successfully employed for treatment of ovarian endometriomas [3]. Single-incision laparoscopic surgery offers the potential for improved surgical cosmesis, decreased postoperative pain and decreased risk of perioperative complications. However, because it requires a single fulcrum for all of the surgical instruments employed, this technique represents the epitome of ergonomic challenge for surgeons [4]. The da Vinci Single-Site (dVSS) platform was approved by the FDA in 2013 to perform hysterectomy and salpingo-oohporectomy with the da Vinci Si Surgical System (Intuitive Surgical, Inc, Sunnyvale, CA) through a single umbilical entry. The dVSS consists of a 2.5 cm silicon single port device with five separate lumens (for insufflation, robotic camera, two robotic instrument arms and an assistant instrument).

The objective of this video article is to describe a safe and reproducible technique of minimally invasive excision and ablation of ovarian endometriomas employing the dVSS platform. Video of procedure. The video is available to download if requested to frpjournal@biomedcentral.com.


## Methods

Twenty-six year old nulligravida with recent diagnosis of large symptomatic bilateral complex ovarian masses, highly suggestive for endometriomas. The patient’s BMI was 23 kg/m2 and had a negative surgical history. The da Vinci Single-Site platform was utilized with 250 mm curved instruments cannulas and the following semi-rigid 5 mm instruments: Maryland Dissector, Monopolar Cautery Hook, Fundus Grasper, Crocodile Grasper and Suction Irrigator. The Maryland Dissector was used to guide a flexible CO_2_ laser fiber (IntelliGuide Laser System, BeamPath flexible fiber and FlexGuide Robotic, Omni Guide, Inc. Cambridge, MA) that was introduced through an 8 mm cannula in the assistant instrument lumen of the single-port device. Our video demonstrates a complete endometrioma stripping in the right ovary and a partial stripping in the left ovary, with CO_2_ laser ablation of the remnant portion of endometrioma overlying the ovarian hilum. Such technique, described by Donnez et al. in conventional multi-port laparoscopy, has been demonstrated to have a minimal impact on ovarian reserve [5]. Complete hemostasis was maintained without the use of electrosurgery on the endometrioma bed. Regenerated oxidized cellulose (Gynecare Interceed, Ethicon US LLC, Cincinnati, OH) was placed around the adnexa at the end of the operation to reduce the chance of postoperative adhesions.

## Results and discussion

Our video illustrates in detail a technique for excision and ablation of large bilateral endometriomas employing single-site technology for with the da Vinci Si surgical system. In particular, we demonstrate the single-site feasibility of a previously described technique of partial stripping and CO_2_ laser ablation. The operation was carried out with minimal mechanical limitations with 5 mm nonwristed semi-rigid instruments. Curved instrument cannulas provide ideal instrument triangulation, and the real-time inversion of laterality provided by the computerized surgical platform makes the surgeon’s movements at the console intuitive and purposeful. This patient underwent an uncomplicated procedure lasting 127 min and was discharged home on the day of surgery. Postoperative course was unremarkable and cosmetic results were excellent.

Novel single incision laparoscopy technology for the da Vinci Si surgical system can be safely applied to the excision and ablation of ovarian endometriomas. Optimal triangulation and inversion of laterality are provided by the system itself, thus the dVSS removes the typical adoption barriers of single-incision laparoscopy [6] and is immediately accessible to surgeons who are proficient in computer-assisted surgery. Use of this technology can be considered in select patients desiring an ultra-minimally invasive approach to ovarian endometriosis, such as young women in reproductive surgery and infertility practices. Of note, because this does not yet constitute an FDA-approved indication for the use of the dVSS (such as hysterectomy and salpingo-oophorectomy), all patients should be specifically informed that this technology will be used, and written consent should be obtained.

## Conclusions

The da Vinci Single-Site platform, currently FDA-approved for hysterectomy and salpingo-oophorectomy, is an enabling tool for minimally invasive conservative ovarian surgery for advanced endometriosis.

## Availability of supporting data

None: this is the first published report on the use of the da Vinci Single-Site platform in ovarian endometriosis.

## Ethical approval

All surgical patients at Brigham and Women’s Hospital sign a detailed informed consent which includes explicit authorization to filming and photographing for teaching and scientific publication purposes when the identity of the patient is safeguarded and not shared.

Brigham and Women’s Hospital exempts researchers from obtaining an IRB approval if less than 4 individual patient data is used for scientific publication purposes. The information in this video-article pertains to a single individual, hence the IRB exemption applies.

## Abbreviations

dVSS: da Vinci Single-Site
FDA: Food and drug administration
CO_2_: Carbon dioxide
